# Modelling the Transmission Dynamics of Tuberculosis in the Ashanti Region of Ghana

**DOI:** 10.1155/2020/4513854

**Published:** 2020-03-31

**Authors:** Felix Okoe Mettle, Prince Osei Affi, Clement Twumasi

**Affiliations:** ^1^Department of Statistics & Actuarial Science, School of Physical and Mathematical Sciences, University of Ghana, Legon, Accra, Ghana; ^2^School of Mathematics, Cardiff University, Cardiff, UK

## Abstract

Mathematical models can aid in elucidating the spread of infectious disease dynamics within a given population over time. In an attempt to model tuberculosis (TB) dynamics among high-burden districts in the Ashanti Region of Ghana, the SEIR epidemic model with demography was employed within both deterministic and stochastic settings for comparison purposes. The deterministic model showed success in modelling TB infection in the region to the transmission dynamics of the stochastic SEIR model over time. It predicted tuberculosis dying out in ten of twelve high-burden districts in the Ashanti Region, but an outbreak in Obuasi municipal and Amansie West district. The effect of introducing treatment at the incubation stage of TB transmission was also investigated, and it was discovered that treatment introduced at the exposed stage decreased the spread of TB. Branching process approximation was used to derive explicit forms of relevant epidemiological quantities of the deterministic SEIR model for stability analysis of equilibrium points. Numerical simulations were performed to validate the overall infection rate, basic reproductive number, herd immunity threshold, and Malthusian parameter based on bootstrapping, jackknife, and Latin Hypercube sampling schemes. It was recommended that the Ghana Health Service should find a good mechanism to detect TB in the early stages of infection in the region. Public health attention must also be given to districts with a potentially higher risk of experiencing endemic TB even though the estimates of the overall epidemic thresholds from our SEIR model suggested that the Ashanti Region as a whole had herd immunity against TB infection.

## 1. Introduction

The burden of tuberculosis (TB) poses a major public health challenge especially among developing countries in terms of its spread. The challenge in controlling TB in Africa is attributed to poverty, drug-resistant tuberculosis, endemic of the causative agents, and inefficient diagnostic methods, among others [1]. TB is considered among the top ten leading causes of deaths worldwide, such that it infects about one-third of the world's population annually according to WHO's health statistics [2]. In 2015 alone, approximately 1.4 million TB-related deaths out of 10.4 million TB cases were recorded globally [3]. The prevalence of the disease is relatively higher in Africa than other parts of the world [4, 5]. In Ghana, the incidence of tuberculosis per 100,000 people was reported at 148 in 2018, with a corresponding mortality rate of 36 [6]. However, less than one-third of the estimated number of diagnosed cases are reported yearly, and the level of under-reporting of diagnosed cases in high TB burden settings is largely unknown [7].

The use of mathematical models and simulations to explore the dynamics of infectious disease has generally gained attention over the years as these models aid in better understanding the spread of such disease infection and the models provide a convenient summary of the epidemiological data [8]. Consequently, it helps to also predict future outbreaks as the infection progresses and make informed decisions for the control of the underlying disease. Models employed to study the transmission of communicable diseases are termed dynamic epidemiological models since they study the evolution of infectious disease over time. Every population may show some degree of heterogeneity about infection, and thus, disease modellers try to incorporate these diversities during the development of mathematical models, resulting in the class of epidemic models known as compartmental models. Compartmental models may be formulated either deterministically using systems of ordinary differential equations (ODEs) or stochastically via continuous-time Markov chains and stochastic differential equations (SDEs). The ODE epidemic model provides a framework for formulating their analogous stochastic models and a major source of comparison with the stochastic epidemic models [9]. Thus, the two types of models are alternative viewpoints of the same infection dynamics [10].

Epidemic processes occur naturally in a stochastic manner especially at the individual level, and thus, stochastic models help to understand that such variations in disease spread may be explained by chance fluctuations alone and not always due to differences in virulence or infectiousness [8]. Nevertheless, stochastic epidemic models are well suited when examining outbreaks in relatively small populations as well as elucidating infection dynamics at early stages [11]. This suggests the robustness of deterministic epidemic models to stochastic perturbations among larger populations. Hence, the deterministic model is suitably an infinite population limit of a general class of stochastic models with or without homogeneous mixing [12, 13]. Hence, we employed a deterministic model for this study due to the size of the target population and lack of information at the early stages of the TB infection within the district, while comparing results with its stochastic version.

In a deterministic model, a heterogeneous population is divided into a finite number of homogeneous subpopulations. Then, the epidemic dynamics are modelled deterministically with a movement among subpopulations using ODEs. Modelling infectious diseases with the deterministic approach has a very long history. These models have been used immensely in studying other communicable diseases such as influenza, chickenpox, measles, and many more in various contexts [14]. Deterministic models provide theoretical results such as the basic reproductive number, thresholds, replacement number, and contact number. In terms of infectious disease, these models help countries, regions, and communities to design proper remedies to reduce the infection probability of the pathogen [15].

Several studies have explored tuberculosis infection from a modelling perspective using different classes of epidemic compartmental models [16]. However, in the process of investigating the effect of exposed individuals on the overall TB infection dynamics of epidemic models, the class of Susceptible-Exposed-Infected-Recovered (SEIR) models is usually employed as compared to the Susceptible-Infected-Recovered (SIR) models which are often adopted generally when the latent stage of the infection is ignored. Most of the studies carried out on TB using the SEIR model explored the global stability with either nonsequential occurrence rate or infectious drive at incubation, infectious, and recovered stages [17, 18]. Nonetheless, only a few studies have been done in Ghana using SEIR models as compared to the SIR model in the context of TB infection since little or no knowledge is known about the exposed individuals within the population at the latent stages of infection. This may be associated with the lack of a proper mechanism to detect exposed individuals and under-reporting in Ghana and other parts of sub-Saharan Africa. However, in the Ashanti Region of Ghana, only one study employed the SEIR model without demography to determine whether or not TB will be endemic in one of the high-burden districts called Amansie West [19]. Their study revealed that TB infection will persist within this district in the Ashanti Region.

Consequently, this current study is an expansion of the previous study [19], by investigating tuberculosis infection dynamics among all high TB burden districts in the Ashanti Region of Ghana using the SEIR model with demography. Here, we developed an SEIR model by incorporating other demographic information such as birth and death to explore the dynamics of TB in the Ashanti Region of Ghana, while examining the effects of (unknown) exposed individuals on the overall infection dynamics within deterministic and stochastic settings for comparison purposes. Moreover, explicit derivation of the basic reproductive number and Malthusian parameter (threshold) was performed using a branching process approximation for steady-state stability analysis. The effects of treatment introduction at the exposed compartment were investigated based on the basic reproductive number. Numerical simulations were performed to validate estimates of the overall infection rate, basic reproductive number, herd immunity threshold, and Malthusian parameter across the districts using estimation techniques from bootstrap, jackknife, and Latin Hypercube sampling schemes for precision.

## 2. Materials and Methods

The study employed administrative data compiled by the Ashanti regional health directorate. The data contained the screening report on tuberculosis in the Ashanti Region of Ghana for the year 2017. Even though there are twenty-seven districts in the Ashanti Region, the data obtained from the health directorate only reported on TB cases within the twelve districts considered as the high-burden districts by the Ghana Health Service. The remaining fifteen districts were seen to produce routine cases of TB in the region. Thus, the infection dynamics of tuberculosis for the entire region was investigated using these high-burden districts. Infected individuals recovered from TB with permanent immunity. Whenever there was infectious contact, it was assumed that the individual goes through the latent (incubation or exposed) period before becoming infectious; hence, we fitted the SEIR model (Susceptible–Exposed-Infected-Recovery model) as originally developed by Kermack and McKendrick [20]. The deterministic SEIR model was employed to study the infection dynamics while comparing its dynamics with its stochastic form. The model was modified by including demography characteristics such as birth and death. Since information on the exposed individuals was not captured or recorded in the actual observed data, a sensitivity analysis was performed on the exposed compartment to examine its effects on the overall TB infection dynamics. Also, the outcome of introducing treatment at the incubation stage of TB transmission was further explored.

From the deterministic SEIR model, the basic reproductive number (*R*_0_) was obtained using the next generation matrix analytically. The population proportion of the compartments at the equilibrium points (disease-free and endemic) was also derived theoretically, and their stability was studied using the Routh–Hurwitz stability criterion. The branching process technique was used to deduce the Malthusian parameter (*ρ*) and the probability of TB extinction. Numerical simulation via bootstrap, jackknife, and Latin Hypercube sampling schemes was carried out to validate the overall empirical infection rate as well as other epidemic thresholds (basic reproductive number, herd immunity threshold, and Malthusian parameter) across the entire region.

### 2.1. Deterministic SEIR Model Development

**Figure 1:**
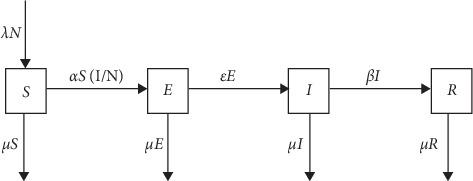
Illustration of the SEIR model.

We present the mathematical formation of the SEIR models with demographic characteristics. The assumption of constant population size is made (demography: birth rate “*λ*” equal to death rate “*μ*”⟺*λ*=*μ*). It is important to note that the notations for the birth (*λ*) and death rates (*μ*) will be maintained to generalize analytical estimators of relevant epidemic quantities from the model for any values of *λ* and *μ*. The deterministic model is formulated by dividing the host population into four classes: Susceptible (*S*), Exposed (*E*), Infectious (*I*), and Recovery (*R*):

(1) $$\begin{array}{c} \text{mathematically}:N=S+E+I+R, \end{array}$$

where *λ*=birth rate, *μ*=death rate, *α*=infection rate, *ε* = rate at which an individual moves from the exposed class to the infection class, and *β* = recovery rate of the infectious individual. The constant population size assumptions together with Figure 1 lead to the following system of ordinary differential equations (ODEs) to indicate the rate of change from one class (disease state) to the other:

(2) $$\begin{array}{c} \frac{\text{d}S}{\text{d}t}=\lambda N-\mu S-\alpha S\frac{1}{N},\\ \frac{\text{d}E}{\text{d}t}=\alpha S\frac{1}{N}-\left(\varepsilon +\mu \right)E,\\ \frac{\text{d}I}{\text{d}t}=\varepsilon E-\left(\beta +\mu \right)I,\\ \frac{\text{d}R}{\text{d}t}=\beta I-\mu R. \end{array}$$

Rescaling equation (2) by representing *s*=(*S*/*N*), *i*=(*I*/*N*),  *r*=(*R*/*N*), and *e*=(*E*/*N*), where *s* = susceptible proportion of the population, *e* = exposed proportion, *i* = infectious proportion, and *r* = recovery proportion [21]. The scaled equations are given as follows:

(3) $$\begin{array}{c} \frac{\text{d}s}{\text{d}t}=\lambda -\mu s-\alpha si,\\ \frac{\text{d}e}{\text{d}t}=\alpha si-\left(\varepsilon +\mu \right)e,\\ \frac{\text{d}i}{\text{d}t}=\varepsilon e-\left(\beta +\mu \right)i,\\ \frac{\text{d}r}{\text{d}t}=\beta i-\mu r, \end{array}$$

where *s*+*e*+*i*+*r*=1. Hence, making “*r*” the subject and setting *r*=1 − *s* − *e* − *i*, it suffices to study the system of ODEs given by equation (4) instead of equation (3):

(4) $$\begin{array}{c} \frac{\text{d}s}{\text{d}t}=\lambda -\mu s-\alpha si,\\ \frac{\text{d}e}{\text{d}t}=\alpha si-\left(\varepsilon +\mu \right)e,\\ \frac{\text{d}i}{\text{d}t}=\varepsilon e-\left(\beta +\mu \right)i. \end{array}$$

This system of ODEs (4) was solved numerically via the standard Runge–Kutta method.

#### 2.1.1. Formulation of the Stochastic SEIR Model with Demography

The stochastic form of the deterministic SEIR model with demography (equation (2)) was constructed by assuming that the TB infection dynamics satisfies a homogeneous continuous-time Markov chain (CTMC) such that time between events or transitions is exponentially distributed. This stochastic model was only developed solely to compare its infection dynamics with the deterministic model. Now, let *S*(*t*), *E*(*t*), *I*(*t*), and *R*(*t*) denote the number of susceptible, exposed, infected, and recovered individuals at any time *t* as the infection progresses. Then, the process {*S*(*t*), *E*(*t*), *I*(*t*), *R*(*t*) : *t* ≥ 0} is CTMC with discrete state space ℱ = {0,1,…, *N*}, where *N*(*t*) = *S*(*t*) + *E*(*t*) + *I*(*t*) + *R*(*t*). Hence, the stochastic SEIR with demography based on its deterministic form can be modelled with events that occur at rates within time interval [*t*, *t* + Δ*t*] according to Table 1.

Gillespie's stochastic simulation algorithm (SSA) [22] was used to simulate the stochastic epidemic SEIR model at 52 time points (in weeks) for over 5000 different simulation runs in parallel. The pseudocode for the simulation according to the continuous-time Markov chain defined is summarized below by Algorithm 1 as implemented in R programming language.

### 2.2. Computation of the Basic Reproductive Number of the SEIR Model Using the Next-Generation Matrix

According to [23], the basic reproductive number (*R*_0_) is the mean number of secondary infections produced by one infective individual in a completely susceptible population at the disease-free equilibrium point (DFEP). Thus, *R*_0_ = (rate of secondary infections) × (infectious period). It was assumed that “*s*” is near the disease-free equilibrium; hence, we linearized the ODEs in equation (4) about the DFEP for exposed and infectious classes yielding the matrix from the next-generation matrix approach [24]:

(5) $$\begin{array}{c} H-K=\left[\begin{array}{cc} 0 & \alpha \\ 0 & 0 \end{array}\right]-\left[\begin{array}{cc} \left(\mu +\varepsilon \right) & 0\\ -\varepsilon & \left(\mu +\beta \right) \end{array}\right], \end{array}$$

where *H* = matrix of infection rates and *K* = matrix of transition rates:

(6) $$\begin{array}{c} H=\left[\begin{array}{cc} 0 & \alpha \\ 0 & 0 \end{array}\right],\\ K=\left[\begin{array}{cc} \left(\mu +\varepsilon \right) & 0\\ -\varepsilon & \left(\mu +\beta \right) \end{array}\right]. \end{array}$$

But

(7) $$\begin{array}{c} \left|K\right|=\left(\mu +\varepsilon \right)\left(\mu +\beta \right)+0⟹\left|K\right|=\left(\mu +\varepsilon \right)\left(\mu +\beta \right),\\ K^{-1}=\frac{1}{\left(\mu +\varepsilon \right)\left(\mu +\beta \right)}\left[\begin{array}{cc} \left(\mu +\beta \right) & 0\\ \varepsilon & \left(\mu +\varepsilon \right) \end{array}\right]. \end{array}$$

Hence,

(8) $$\begin{array}{c} K^{-1}=\left[\begin{array}{cc} \frac{1}{\left(\mu +\varepsilon \right)} & 0\\ \frac{\varepsilon }{\left(\mu +\varepsilon \right)\left(\mu +\beta \right)} & \frac{1}{\left(\mu +\beta \right)} \end{array}\right]. \end{array}$$

Multiplying the inverse of “*K*” by the matrix “*H*” results in

(9) $$\begin{array}{c} HK^{-1}=\left[\begin{array}{cc} 0 & \alpha \\ 0 & 0 \end{array}\right]\left[\begin{array}{cc} \frac{1}{\left(\mu +\varepsilon \right)} & 0\\ \frac{\varepsilon }{\left(\mu +\varepsilon \right)\left(\mu +\beta \right)} & \frac{1}{\left(\mu +\beta \right)} \end{array}\right]=\left[\begin{array}{cc} \frac{\alpha \varepsilon }{\left(\mu +\varepsilon \right)\left(\mu +\beta \right)} & \frac{\alpha }{\left(\mu +\beta \right)}\\ 0 & 0 \end{array}\right]. \end{array}$$

The basic reproductive number (*R*_0_) is also defined as the spectral radius of *HK*^−1^ according to [24]. We denote this by *ρ*(*HK*^−1^); hence,

(10) $$\begin{array}{c} R_{0}=\frac{\alpha \varepsilon }{\left(\beta +\mu \right)\left(\varepsilon +\mu \right)}. \end{array}$$

**Remark 1 .**

It is important to note that *R*_0_ as used here is a natural bifurcation parameter at 1 such that *R*_0_ < 1 implies the infection will die out in the long run and persist in the population if otherwise (*R*_0_ > 1). Given *R*_0_, the herd immunity or critical immunization threshold (*q*_*c*_) which measures the proportion of the population that needs to be immunized to control the infection transmission is estimated as [25]

(11) $$\begin{array}{c} q_{c}=1-\frac{1}{R_{0}}. \end{array}$$

### 2.3. Equilibrium Point of the SEIR Model

For the purpose of this study, we considered two equilibrium points: the disease-free equilibrium point (DFEP) when *i*=0 and the endemic equilibrium point (EEP) when *i* ≠ 0. To achieve this, equation (4) was set to zero and then the values of *s*, *e*,  and *i* were determined analytically:

(12) $$\begin{array}{c} \frac{\text{d}s}{\text{d}t}=0⟹\lambda -\mu s-\alpha si=0,\\ \frac{\text{d}e}{\text{d}t}=0⟹\alpha si-\left(\varepsilon +\mu \right)e=0,\\ \frac{\text{d}i}{\text{d}t}=0⟹\varepsilon e-\left(\beta +\mu \right)i=0. \end{array}$$

#### 2.3.1. Disease-Free Equilibrium Point

At the disease-free equilibrium point, it is presumed that there is no infection or disease in the system; that is, *i*=0 and *e*=0. Hence, from equation (12), we obtain

(13) $$\begin{array}{c} \lambda -\mu s-\alpha s\left(0\right)=0,\\ \alpha s\left(0\right)-\left(\varepsilon +\mu \right)\left(0\right)=0,\\ \varepsilon \left(0\right)-\left(\beta +\mu \right)\left(0\right)=0. \end{array}$$

These equations at the DFE reduce to

(14) $$\begin{array}{c} \lambda -\mu s=0,\\ \lambda =\mu s⟹\frac{\lambda }{\mu }. \end{array}$$

Therefore, at the DFE, (*s*, *e*, *i*)={(*λ*/*μ*), 0,0}=(1,0,0) since the host population is constant (*λ*=*μ*).

#### 2.3.2. Endemic Equilibrium Point

At the endemic equilibrium point, disease persists in the system at the steady state. Here, we solve equation (12) to obtain *s*, *e*, and *i*. But for easy identification, *s*, *e*,  and *i* are represented by (*s*^*∗*^, *e*^*∗*^, *i*^*∗*^) as population proportion of the compartments at the steady state.

From

(15) $$\begin{array}{c} \varepsilon e-\left(\mu +\beta \right)i=0⟹i=\frac{\varepsilon e}{\mu +\beta },\\ \alpha si-\left(\mu +\varepsilon \right)e=0⟹s=\frac{\left(\mu +\varepsilon \right)}{\alpha i}. \end{array}$$

Putting *i* into *s* gives

(16) $$\begin{array}{c} s=\frac{\left(\mu +\varepsilon \right)e}{\alpha \left(\varepsilon e/\left(\mu +\beta \right)\right)}=\frac{\left(\mu +\varepsilon \right)\left(\mu +\beta \right)e}{\alpha \varepsilon e}. \end{array}$$

Thus,

(17) $$\begin{array}{c} s^{*}=\frac{\left(\mu +\varepsilon \right)\left(\mu +\beta \right)}{\alpha \varepsilon }. \end{array}$$

Also, from *αsi* − (*μ*+*ε*)*e*=0, we have *αsi*=(*μ*+*ε*)*e* and putting this into

(18) $$\begin{array}{c} \lambda -\mu s-\alpha si=0,\;\mathrm{yields}\;\lambda -\mu s-\left(\mu +\varepsilon \right)e=0,\\ \lambda -\mu s+\left(-\mu -\varepsilon \right)e=0,\\ \left(-\mu -\varepsilon \right)e=-\lambda +\mu s. \end{array}$$

Substituting *s*^*∗*^ into (18) gives

(19) $$\begin{array}{c} -\left(\mu +\varepsilon \right)e=\left\{\lambda -\mu \frac{\left(\mu +\varepsilon \right)\left(\mu +\beta \right)}{\alpha \varepsilon }\right\}, \end{array}$$

where

(20) $$\begin{array}{c} e^{*}=\frac{\lambda \alpha \varepsilon -\mu \left(\mu +\varepsilon \right)\left(\mu +\beta \right)}{\alpha \varepsilon \left(\mu +\varepsilon \right)}. \end{array}$$

Putting *e*^*∗*^ into *i*=(*εe*/(*μ*+*β*)) yields

(21) $$\begin{array}{c} i^{*}=\frac{\lambda \alpha \varepsilon -\mu \left(\mu +\varepsilon \right)}{\alpha \left(\mu +\varepsilon \right)}. \end{array}$$

Hence, at the endemic equilibrium point,

(22) $$\begin{array}{c} \left(s^{*},e^{*},i^{*}\right)=\left\{\frac{\left(\mu +\varepsilon \right)\left(\mu +\beta \right)}{\alpha \varepsilon },\frac{\lambda \alpha \varepsilon -\mu \left(\mu +\varepsilon \right)\left(\mu +\beta \right)}{\alpha \varepsilon \left(\mu +\varepsilon \right)},\frac{\lambda \alpha \varepsilon -\mu \left(\mu +\varepsilon \right)}{\alpha \left(\mu +\varepsilon \right)}\right\}. \end{array}$$

#### 2.3.3. Stability of the Disease-Free Equilibrium Point

The stability of disease-free equilibrium point was also determined based on Theorem 1.

**Theorem 1 .**

The disease-free equilibrium point of system (4) is asymptotically stable if and only if *R*_0_ < 1 and unstable if *R*_0_ > 1.

**Proof**

At the DFE, we obtained the Jacobian matrix about the point (*s*, *e*, *i*)=(1,0,0). This yields the following matrix:

(23) $$\begin{array}{c} J\left(s,e,i\right)=\left[\begin{array}{ccc} -\mu & 0 & -\alpha \\ 0 & -\left(\mu +\varepsilon \right) & \alpha \\ 0 & \varepsilon & -\left(\mu +\beta \right) \end{array}\right]. \end{array}$$

We let *J*(*s*, *e*, *i*)_*DFE*_=*J*(*s*, *e*, *i*) be the Jacobian matrix at the disease-free equilibrium and solve the characteristics equation of *J*(*s*, *e*, *i*)_*DFE*_. This can be achieved by solving the relation: *J*(*s*, *e*, *i*)_*DFE*_ − *Iλ*, where *I* is a unit matrix with order 3 by 3 since *J*(*s*, *e*, *i*)_*DFE*_ has the same order:

(24) $$\begin{array}{c} I\lambda =\left[\begin{array}{ccc} 1 & 0 & 0\\ 0 & 1 & 0\\ 0 & 0 & 1 \end{array}\right]=\left[\begin{array}{ccc} \lambda & 0 & 0\\ 0 & \lambda & 0\\ 0 & 0 & \lambda \end{array}\right],\\ J{\left(s,e,i\right)}_{DFE}-I\lambda =\left[\begin{array}{ccc} -\mu & 0 & -\alpha \\ 0 & -\left(\mu +\varepsilon \right) & \alpha \\ 0 & \varepsilon & -\left(\mu +\beta \right) \end{array}\right]-\left[\begin{array}{ccc} \lambda & 0 & 0\\ 0 & \lambda & 0\\ 0 & 0 & \lambda \end{array}\right],\\ J{\left(s,e,i\right)}_{DFE}-I\lambda =\left[\begin{array}{ccc} -\left(\mu +\lambda \right) & 0 & -\alpha \\ 0 & -\left\{\left(\mu +\varepsilon \right)+\lambda \right\} & \alpha \\ 0 & \varepsilon & -\left\{\left(\mu +\varepsilon \right)+\lambda \right\} \end{array}\right]. \end{array}$$

From this, we obtained the characteristics equation of *J*(*s*, *e*, *i*)_*DFE*_ − *Iλ* by finding the determinant and equating it to zero:

(25) $$\begin{array}{c} \left|J{\left(s,e,i\right)}_{DFE}-I\lambda \right|=\left|\begin{array}{ccc} -\left(\mu +\lambda \right) & 0 & -\alpha \\ 0 & -\left\{\left(\mu +\varepsilon \right)+\lambda \right\} & \alpha \\ 0 & \varepsilon & -\left\{\left(\mu +\varepsilon \right)+\lambda \right\} \end{array}\right|. \end{array}$$

For |*J*(*s*, *e*, *i*)_*DFE*_ − *Iλ*|=0, we have

(26) $$\begin{array}{c} \lambda^{3}+\left(3\mu +\varepsilon +\beta \right)\lambda^{2}+\left(3\mu^{2}+2\mu \beta +2\varepsilon \mu +\varepsilon \beta -\alpha \varepsilon \right)\lambda +\left(\mu^{2}+\mu^{2}\beta +\mu^{2}\varepsilon +\mu \varepsilon \beta -\mu \alpha \varepsilon \right)=0. \end{array}$$

Letting *Y* and *Z* be the coefficients of *λ*^2^ and *λ*, respectively, and *A* be the constant term of equation (26); then,

(27) $$\begin{array}{c} Y=3\mu +\varepsilon +\beta ,\\ Z=3\mu^{2}+2\mu \beta +2\varepsilon \mu +\varepsilon \beta -\alpha \varepsilon ,\\ A=\mu^{2}+\mu^{2}\beta +\mu^{2}\varepsilon +\mu \varepsilon \beta -\mu \alpha \varepsilon . \end{array}$$

The characteristic equation becomes *λ*^3^+*Yλ*^2^+*Zλ*+*A*.

From Routh–Hurwitz stability criterion analysis, if *Y* > 0,  *Z* > 0, and *YZ* − *A* > 0, then, all the roots of the characteristic equation have a negative real part; hence, the equilibrium point (DFEP) is stable.

#### 2.3.4. Stability of the Endemic Equilibrium Point

Theorem 2 was employed to determine the stability of the endemic equilibrium point.

**Theorem 2 .**

The endemic equilibrium of system (4) is also asymptotically stable when *R*_0_ > 1 and unstable when *R*_0_ < 1.

**Proof**

Obtaining the Jacobian matrix about the point (*s*^*∗*^, *e*^*∗*^, *i*^*∗*^) gives

(28) $$\begin{array}{c} J\left(s^{*},e^{*},i^{*}\right)=\left[\begin{array}{ccc} -\mu -\alpha i^{*} & 0 & -\alpha s^{*}\\ \alpha i^{*} & -\left(\mu +\varepsilon \right) & \alpha s^{*}\\ 0 & \varepsilon & -\left(\mu +\beta \right) \end{array}\right]. \end{array}$$

Let *J*(*s*^*∗*^, *e*^*∗*^, *i*^*∗*^)_*EEP*_ represent the Jacobian matrix at the endemic equilibrium point. We solve the characteristic equation of *J*(*s*^*∗*^, *e*^*∗*^, *i*^*∗*^)_*EEP*_ by finding the determinant of *J*(*s*^*∗*^, *e*^*∗*^, *i*^*∗*^)_*EEP*_ − *Iλ* and setting the results to zero:

(29) $$\begin{array}{c} I\lambda =\left[\begin{array}{ccc} 1 & 0 & 0\\ 0 & 1 & 0\\ 0 & 0 & 1 \end{array}\right]=\left[\begin{array}{ccc} \lambda & 0 & 0\\ 0 & \lambda & 0\\ 0 & 0 & \lambda \end{array}\right],\\ J{\left(s^{*},e^{*},i^{*}\right)}_{EEP}-I\lambda =\left[\begin{array}{ccc} -\mu -\alpha i^{*} & 0 & -\alpha s^{*}\\ \alpha i^{*} & -\left(\mu +\varepsilon \right) & \alpha s^{*}\\ 0 & \varepsilon & -\left(\mu +\beta \right) \end{array}\right]-\left[\begin{array}{ccc} \lambda & 0 & 0\\ 0 & \lambda & 0\\ 0 & 0 & \lambda \end{array}\right],\\ J{\left(s^{*},e^{*},i^{*}\right)}_{EEP}-I\lambda =\left[\begin{array}{ccc} -\left(\mu +\alpha i^{*}+\lambda \right) & 0 & -\alpha s^{*}\\ \alpha i^{*} & -\left(\mu +\varepsilon +\lambda \right) & \alpha s^{*}\\ 0 & \varepsilon & -\left(\mu +\beta +\lambda \right) \end{array}\right]. \end{array}$$

Finding the characteristics equation of *J*(*s*^*∗*^, *e*^*∗*^, *i*^*∗*^)_*EEP*_ − *Iλ*, we find the determinant of *J*(*s*^*∗*^, *e*^*∗*^, *i*^*∗*^)_*EEP*_ − *Iλ* and set it to zero:

(30) $$\begin{array}{c} \left|J{\left(s^{*},e^{*},i^{*}\right)}_{EEP}-I\lambda \right|=\left|\begin{array}{ccc} -\left(\mu +\alpha i^{*}+\lambda \right) & 0 & -\alpha s^{*}\\ \alpha i^{*} & -\left(\mu +\varepsilon +\lambda \right) & \alpha s^{*}\\ 0 & \varepsilon & -\left(\mu +\beta +\lambda \right) \end{array}\right|. \end{array}$$

Hence, |*J*(*s*^*∗*^, *e*^*∗*^, *i*^*∗*^)_*EEP*_ − *Iλ*|=0 results in

(31) $$\begin{array}{c} \lambda^{3}+\left(3\mu +\alpha i^{*}+\varepsilon +\beta \right)\lambda^{2}+\left(2\mu^{2}+2\mu \varepsilon +\mu \beta +2\alpha i^{*}\mu +\alpha i^{*}\varepsilon +\alpha i^{*}\beta +\varepsilon \beta -\varepsilon \alpha s^{*}\right)\lambda \\ +\left(\mu^{2}+\varepsilon \mu^{2}+\mu \varepsilon \beta -\mu \varepsilon \alpha s^{*}+\alpha i^{*}\mu^{2}+\alpha i^{*}\varepsilon \mu +\alpha i^{*}\varepsilon \beta \right)=0. \end{array}$$

We let *Y* and *Z* represent the coefficient of *λ*^2^ and *λ*, respectively, and let *A* be the constant term in equation (31). Hence,

(32) $$\begin{array}{c} Y=3\mu +\alpha i^{*}+\varepsilon +\beta ,\\ Z=2\mu^{2}+2\mu \varepsilon +\mu \beta +2\alpha i^{*}\mu +\alpha i^{*}\varepsilon +\alpha i^{*}\beta +\varepsilon \beta -\varepsilon \alpha s^{*},\\ A=\mu^{2}+\varepsilon \mu^{2}+\mu \varepsilon \beta -\mu \varepsilon \alpha s^{*}+\alpha i^{*}\mu^{2}+\alpha i^{*}\varepsilon \mu +\alpha i^{*}\varepsilon \beta . \end{array}$$

The characteristics equation of *J*(*s*^*∗*^, *e*^*∗*^, *i*^*∗*^)_*EEP*_ − *Iλ* then becomes *λ*^3^+*Yλ*^2^+*Zλ*+*A*=0. Using the Routh–Hurwitz stability analysis and assuming *Y* > 0,  *Z* > 0, and *YZ* − *A* > 0, all the zeros of the characteristics equation have a negative real part; hence, the equilibrium (endemic) point is stable.

### 2.4. Deterministic Formulation of the SEIR Model with the Introduction of Treatment at the Exposed Stage

In the formulation of the SEIR model with introduction of treatment at the exposed (incubation) stage, the assumptions made were as follows: there is a constant population size, the exposed individuals are not infectious, the exposed individuals receive treatment, and finally, the exposed individuals may either recover the susceptible compartment, may die, or become infectious.

Similarly, *λ* = birth rate, *µ* = death rate, *α* = is the infection rate, *ε* = the rate at which an individual moves from the exposed class to the infection class, *τ*  = treatment rate introduced at the exposed stage, and *β* = recovery rate of the infectious individual. The SEIR model with treatment at the latent stage is primarily considered in this study to obtain an estimator for *R*_0_ based on its systems of ODEs. Consequently, the effects of treatment introduction in the exposed compartment on the *R*_0_ are only examined.

#### 2.4.1. Mathematical Formulation of the SEIR Model with the Introduction of Treatment at the Exposed Stage

**Figure 2:**
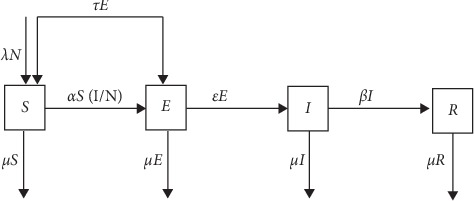
Illustration of the SEIR model with the introduction of treatment at the exposed stage.

This model as the standard SEIR model has the host population divided into four compartments which are Susceptible (*S*), Exposed (*E*), Infected (*I*), and Recovery (*R*).  Figure 2, together with assumptions made, shows the following system of ODEs representing the model by the following equation:

(33) $$\begin{array}{c} \frac{\text{d}S}{\text{d}t}=\lambda N-\mu S-\alpha S\frac{1}{N}+\tau E,\\ \frac{\text{d}E}{\text{d}t}=\alpha S\frac{1}{N}-\left(\tau +\varepsilon +\mu \right)E,\\ \frac{\text{d}I}{\text{d}t}=\varepsilon E-\left(\beta +\mu \right)I,\\ \frac{\text{d}R}{\text{d}t}=\beta I-\mu R. \end{array}$$

Rescaling equation (33), we represent *s*=(*S*/*N*),  *e*=(*E*/*N*),  *i*=(*I*/*N*), and *r*=(*R*/*N*), where *s*, *e*, *i*, and *r* represent the susceptible, exposed, infectious, and recovery/removal population proportions, respectively [21]. Substituting the proportions into equation (33) results in

(34) $$\begin{array}{c} \frac{\text{d}s}{\text{d}t}=\lambda -\mu s-\alpha si+\tau e,\\ \frac{\text{d}e}{\text{d}t}=\alpha si-\left(\tau +\varepsilon +\mu \right)e,\\ \frac{\text{d}i}{\text{d}t}=\varepsilon e-\left(\beta +\mu \right)i,\\ \frac{\text{d}r}{\text{d}t}=\beta i-\mu r, \end{array}$$

where *s*+*e*+*i*+*r*=1. Hence, making “*r*” the subject and setting *r*=1 − *s* − *e* − *i*, it is enough to study the system in (35) instead of equation (34):

(35) $$\begin{array}{c} \frac{\text{d}s}{\text{d}t}=\lambda -\mu s-\alpha si+\tau e,\\ \frac{\text{d}e}{\text{d}t}=\alpha si-\left(\tau +\varepsilon +\mu \right)e,\\ \frac{\text{d}i}{\text{d}t}=\varepsilon e-\left(\beta +\mu \right)i. \end{array}$$

#### 2.4.2. Computation of the Basic Reproductive Number of the SEIR Model with Treatment Introduction at the Exposed Stage

*R*_0_ = (rate of secondary infections) × (duration of infection); hence, linearizing equation (35) leads to the next-generation matrix:

(36) $$\begin{array}{c} H-K=\left[\begin{array}{cc} 0 & \alpha \\ 0 & 0 \end{array}\right]-\left[\begin{array}{cc} \left(\tau +\mu +\varepsilon \right) & 0\\ -\varepsilon & \left(\mu +\beta \right) \end{array}\right]. \end{array}$$

where *H* = matrix of infection rates and *K* = matrix of transition rates:

(37) $$\begin{array}{c} H=\left[\begin{array}{cc} 0 & \alpha \\ 0 & 0 \end{array}\right],\\ K=\left[\begin{array}{cc} \left(\tau +\mu +\varepsilon \right) & 0\\ -\varepsilon & \left(\mu +\beta \right) \end{array}\right]. \end{array}$$

But, |*K*|=(*τ*+*μ*+*ε*)(*μ*+*β*)+0⟹|*K*|=(*τ*+*μ*+*ε*)(*μ*+*β*):

(38) $$\begin{array}{c} K^{-1}=\frac{1}{\left(\tau +\mu +\varepsilon \right)\left(\mu +\beta \right)}\left[\begin{array}{cc} \left(\mu +\beta \right) & 0\\ \varepsilon & \left(\tau +\mu +\varepsilon \right) \end{array}\right]. \end{array}$$

Hence, $K^{-1}=\left[\begin{array}{cc} 1/\left(\tau +\mu +\varepsilon \right) & 0\\ \varepsilon /\left(\left(\tau +\mu +\varepsilon \right)\left(\mu +\beta \right)\right) & 1/\left(\mu +\beta \right) \end{array}\right]$.

Multiplying the inverse of “*K*” by the matrix “*H*” results in

(39) $$\begin{array}{c} HK^{-1}=\left[\begin{array}{cc} 0 & \alpha \\ 0 & 0 \end{array}\right]\left[\begin{array}{cc} \frac{1}{\left(\tau +\mu +\varepsilon \right)} & 0\\ \frac{\varepsilon }{\left(\tau +\mu +\varepsilon \right)\left(\mu +\beta \right)} & \frac{1}{\left(\mu +\beta \right)} \end{array}\right]=\left[\begin{array}{cc} \frac{\alpha \varepsilon }{\left(\tau +\mu +\varepsilon \right)\left(\mu +\beta \right)} & \frac{\alpha }{\left(\mu +\beta \right)}\\ 0 & 0 \end{array}\right]. \end{array}$$

Thus,

(40) $$\begin{array}{c} R_{0}=\frac{\alpha \varepsilon }{\left(\beta +\mu \right)\left(\tau +\mu +\varepsilon \right)}. \end{array}$$

### 2.5. Branching Process Approximation of the Epidemic Process

In the early stage of the epidemics, the infection rate is relatively small. The exposed class at any time (*E*(*t*) is assigned a rate (*αI*(*t*)*S*(*t*)/*N*(*t*)) and is being reduced by the rate (*ε*+*μ*)*E*(*t*). The infectious class population, on the other hand, is increased by the rate *εE*(*t*) and reduced by (*β*+*μ*)*I*(*t*). At the initial stage, the host population *N*(*t*) is almost the same as the susceptible population *S*(*t*); hence, the ratio of the two is approximately one (*S*(*t*)/*N*(*t*)≃1). This implies that the exposed class tends to be increased at the rate *αI*(*t*) instead of (*αI*(*t*)*S*(*t*)/*N*(*t*)) [26].

We let *T*_*n*_(*t*)=*E*_*n*_(*t*)+*I*_*n*_(*t*) denote the number of infected individuals at time *t*. From this relation, *T*_*n*_(*t*) is approximated by the branching process according to Theorem 3.

**Theorem 3 .**

If *T*_*n*_(*t*) is an epidemic process and *T*_*∞*_(*t*) is the branching process, then *T*_*n*_(*t*) converges weakly to *T*_*∞*_(*t*), that is, *T*_*n*_⟹*T*_*∞*_,  *n*⟶*∞* on any finite interval [0, *t*_1_].

The approximation from Theorem 3 has two stages, namely, the childhood (exposed) *E*_*∞*_ and the adulthood (infectious)*I*_*∞*_ [27]. At the initial stage of the process where *t*=0, (*E*_*∞*_(0), *I*_*∞*_(0))=(1,0); *E*_*∞*_(*t*) is increased by *αI*_*∞*_(*t*) and reduced by rate (*μ*+*ε*)*E*_*∞*_(*t*). *I*_*∞*_(*t*) is increased by *εE*_*∞*_(*t*) (end of childhood) and reduced by (*μ*+*β*)*I*_*∞*_(*t*) (end of adulthood). However, when state *I*(*t*) reaches the absorbing state, the disease (TB) transmission stops.

#### 2.5.1. Computation of the Malthusian Parameter

We derive the threshold *ρ* (Malthusian parameter) from the branching process *T*_*∞*_.

Malthusian parameter is the intrinsic exponential growth rate of the epidemic branching process (*T*_*∞*_). The spread of the epidemic stops when the Malthusian parameter is less than zero (*ρ* < 0). We denote it by *ρ*; hence,

(41) $$\begin{array}{c} \int_{0}^{\infty }e^{-\rho t}g\left(t\right)\text{d}t=1, \end{array}$$

where *g*(*t*) represents the average rate at which an individual gives birth (infectious contact) at time *t* [28].

**Theorem 4 .**

The Malthusian parameter of the epidemic is given as

(42) $$\begin{array}{c} \rho =-\left\{\mu +\frac{\varepsilon +\beta }{2}\right\}+\sqrt{\frac{{\left(\varepsilon -\beta \right)}^{2}}{4}+\alpha \varepsilon }. \end{array}$$

**Proof**

At the end of the exposed period, there is no contact, but *α* contact rate at the infectious period gives

(43) $$\begin{array}{c} g\left(t\right)=\alpha e^{-\mu t}\int_{0}^{t}\varepsilon e^{-\varepsilon s}e^{-\beta \left(t-s\right)}\text{d}s⟹g\left(t\right)=\alpha \varepsilon e^{-\left(\mu +\beta \right)t}\int_{0}^{t}e^{-\left(\varepsilon -\beta \right)s}\text{d}s. \end{array}$$

Integrating *g*(*t*) with respect to *s* and applying the limit gives

(44) $$\begin{array}{c} g\left(t\right)=\left\{\begin{array}{cc} \frac{\alpha \varepsilon }{\varepsilon -\beta }\left(e^{-\left(\mu +\beta \right)t}-e^{-\left(\varepsilon +\mu \right)t}\right), & \mathrm{if}\;\varepsilon \neq \beta ,\\ \alpha \varepsilon te^{-\left(\mu +\beta \right)}, & \mathrm{if}\;\varepsilon =\beta . \end{array}\right. \end{array}$$

Substituting *g*(*t*) obtained above into (12) gives

(45) $$\begin{array}{c} \left\{\begin{array}{cc} -\left\{\mu +\frac{\varepsilon +\beta }{2}\right\}+\sqrt{\frac{{\left(\varepsilon -\beta \right)}^{2}}{4}+\alpha \varepsilon }, & \mathrm{if}\;\varepsilon \neq \beta ,\\ \sqrt{\alpha \varepsilon }-\left(\mu +\beta \right), & \mathrm{if}\;\varepsilon =\beta . \end{array}\right. \end{array}$$

By considering a situation where *ε* ≠ *β*, we have

(46) $$\begin{array}{c} \rho =-\left\{\mu +\frac{\varepsilon +\beta }{2}\right\}+\sqrt{\frac{{\left(\varepsilon -\beta \right)}^{2}}{4}+\alpha \varepsilon }. \end{array}$$

#### 2.5.2. Probability of Disease (Tuberculosis) Extinction

The probability of extinction is derived using the branching process approximation of the epidemic process. Both the probabilities of extinction of the epidemic when started with one latent Π(1,0) individual and when started with one infectious Π(0,1) individual are derived as two points. Also, the probability of extinction of the epidemic when it starts with “*n*” latent and “*z*” infectious individuals using the two points Π(1,0) and Π(0,1) were deduced. To derive these two points, we assumed geometric offspring probability generating function as proposed by Lloyd and others [29]. We let “Π” be the smallest positive solution of the equation *q*=*f*(*q*) assuming one incubated individual, with *f* denoting the probability generating function of *X*. Then,

(47) $$\begin{array}{c} f\left(q\right)=\overset{\infty }{\underset{r=0}{\sum }}P\left(X=r\right)q^{r}\\ =\frac{\mu }{\mu +\varepsilon }\frac{\varepsilon }{\mu +\varepsilon }\frac{\beta +\mu }{\alpha +\beta +\mu }+\overset{\infty }{\underset{r=1}{\sum }}\frac{\varepsilon }{\mu +\varepsilon }\frac{\beta +\mu }{\alpha +\beta +\mu }{\left(\frac{r}{\alpha +\beta +\mu }\right)}^{r}q^{r}\\ =A+\frac{\left(1-A\right)B}{1-\left(1-B\right)\mu },\;\mathrm{where}\;A=\frac{\mu }{\mu +\varepsilon }\;\mathrm{and}\;B=\frac{\beta +\mu }{\alpha +\beta +\mu }. \end{array}$$

However, “Π” is the smallest solution in the range [0,1] of the following equation:

(48) $$\begin{array}{c} q=A+\frac{\left(1-A\right)B}{1-\left(1-B\right)\mu }. \end{array}$$

Equation (48) has two solutions: *q*_*o*_=1 and *q*_1_=*A*+(*B*/1 − *B*):

(49) $$\begin{array}{c} q_{1}=\frac{\mu }{\mu +\varepsilon }\frac{\varepsilon }{\mu +\varepsilon }\frac{\beta +\mu }{\alpha }=\frac{\mu }{\mu +\varepsilon }\frac{\varepsilon }{\mu +\varepsilon }\frac{1}{R_{0}}. \end{array}$$

This yields the following two points:

(50) $$\begin{array}{c} \Pi \left(1,0\right)=\left\{\begin{array}{cc} 1, & \mathrm{if}\;R_{0}\leq 1,\\ \frac{\mu }{\mu +\varepsilon }+\frac{\varepsilon }{\mu +\varepsilon }\frac{1}{R_{0}}, & \mathrm{if}\;R_{0}>1, \end{array}\right.\\ \Pi \left(0,1\right)=\left\{\begin{array}{cc} 1, & \mathrm{if}\;R_{0}\leq 1,\\ \frac{1}{R_{0}}, & \mathrm{if}\;R_{0}>1. \end{array}\right. \end{array}$$

Therefore, the probability of disease extinction in general is

(51) $$\begin{array}{c} \Pi \left(n,z\right)={\left[\Pi \left(1,0\right)\right]}^{n}{\left[\Pi \left(0,1\right)\right]}^{z}, \end{array}$$

since all the *n*+*z* independent epidemics must die out as suggested by Lahodny and others [30]. This results in

(52) $$\begin{array}{c} \Pi \left(n,z\right)=\left\{\begin{array}{cc} 1, & \mathrm{if}\;R_{0}\leq 1,\\ {\left(\frac{\mu }{\mu +\varepsilon }+\frac{\varepsilon }{\mu +\varepsilon }\frac{1}{R_{0}}\right)}^{n}{\left(\frac{1}{R_{0}}\right)}^{z}, & \mathrm{if}\;R_{0}>1. \end{array}\right. \end{array}$$

## 3. Results

### 3.1. SEIR Model Parameter Estimation

The parameters of the SEIR model with demography as used in this study include birth (*λ*) and death (*μ*) rates, the infection rate (*α*), exposed rate (*ε*), and finally, the recovery rate (*β*). These parameters were estimated from different sources. Some were computed from the data from the regional health directorate and others from the literature. Birth (*λ*) and death (*μ*) rates and the recovery rate (*β*) were estimated from the existing literature on TB infection. The natural death rate in Ghana is estimated to be 7 deaths per 1000 individuals [31]. But due to the assumption of a closed population system, the natural birth and death rates are assumed to be equal; hence, *λ*=*μ*=0.007. Also, the average period of infection is simply the reciprocal of the recovery rate as defined in [32]. TB has an average infectious period of two weeks [33, 34]. Thus,

(53) $$\begin{array}{c} \beta =\frac{1}{\text{infectious period}}=\frac{1}{2}=0.5\;\mathrm{per}\;\mathrm{week}. \end{array}$$

Moreover, TB is said to have an average exposed period of six weeks. Hence, the exposed rate is estimated as

(54) $$\begin{array}{c} \varepsilon =\frac{1}{\text{average exposed period}}=\frac{1}{6}=0.1667. \end{array}$$

The infection rate (*α*) was estimated from the actual TB data obtained from the regional health directorate as proposed in [34]:

(55) $$\begin{array}{c} \alpha =\frac{\text{effective contact}}{\text{total contact}}. \end{array}$$

Table 2 shows the total contact and effective contact for each of the twelve high-burden districts with their corresponding estimates of the infection rates. Nonetheless, the recovery rate, natural birth, and death rate estimates were assumed equal across the districts.

### 3.2. Estimate of the Basic Reproductive Number, Herd Immunity Threshold, and the Malthusian Parameter

Based on the empirical data, it was found that the estimates of the basic reproductive number, herd immunity threshold, and Malthusian parameter across the entire region are *R*_0_=0.518 < 1, *q*_*c*_=−0.9305 < 0, and (*ρ*)=−0.074 < 0, respectively (Table 3). Thus, the probability of TB extinction (Π) in the entire region based on the branching process estimation is 1 since overall *R*_0_ < 1 (from equation (48)). The estimates of these epidemic thresholds imply that TB infection is expected to yield extinction in the entire Ashanti Region of Ghana, and thus, the disease-free equilibrium point will be stable. It was revealed that Amansie West and Obuasi Municipal were the only districts among the high-burden districts with *R*_0_ > 1, *q*_*c*_ > 0, and *ρ* > 0 (see Table 2). Hence, TB infection is expected to persist in these districts resulting in the endemic equilibrium point. From the herd immunity threshold estimates of these two districts with a risk of endemic TB, approximately 14% of Amansie West population and 12% of Obuasi Municipal need to be immunized to control the spread of the disease, respectively. However, estimates of the epidemic thresholds for the remaining ten districts suggest that TB infection will not persist over time.

### 3.3. Stability Analysis of the Equilibrium Points across the High-Burden District and the Entire Region

The stability analysis of the equilibrium points of the SEIR deterministic model with demographic characteristics for each of the 12 high-burden districts in the Ashanti Region was investigated based on Theorem 1, Theorem 2, and the Routh–Hurwitz stability criterion. The basic reproductive number for each of the districts indicated that some of the districts are characterized by the disease-free equilibrium point and others, the endemic equilibrium point. Also, from the Routh–Hurwitz stability criterion for the disease-free equilibrium point, the characteristics equation was derived as *λ*^3^+*Yλ*^2^+*Zλ*+*A*. The values of coefficients *Y*, *Z*, *A*, and *YZ* − *A* of the characteristics equation for the disease-free districts are, respectively, summarized in Table 4. On the other hand, Table 5 presents the values of the coefficients of the underlying characteristics equation for the two districts that resulted in endemic cases.

From Table 4, it was revealed that all the conditions for the stability of the disease-free equilibrium point hold according to the Routh–Hurwitz stability criterion: all *Y* > 0, all *Z* > 0, and all *YZ* − *A* > 0; hence, the equilibrium point of the 10 districts characterized by disease-free cases is stable. Similar to the disease-free cases, the endemic cases have the equilibrium point stable since (from Table 5) the Routh–Hurwitz stability criterion conditions hold for the other 2 districts. For the entire region under study, the disease-free equilibrium point was stable according to the basic reproductive number and Theorem 1. By the Routh-Hurwitz stability analysis, the characteristics equation deduced for the disease-free equilibrium had the following coefficients: *Y*=0.6877,  *Z*=3.04716,  *A*=0.0003461, and *YZ* − *A*=2.095186. Also, *Y* > 0,  *Z* > 0, and *YZ* − *A* > 0 indicate that all the zeros of the characteristics equation will have a negative real part; and since all these conditions hold, the disease-free equilibrium point of the entire region is thus stable.

### 3.4. Sensitivity Analysis of the SEIR Model

**Figure 3:**
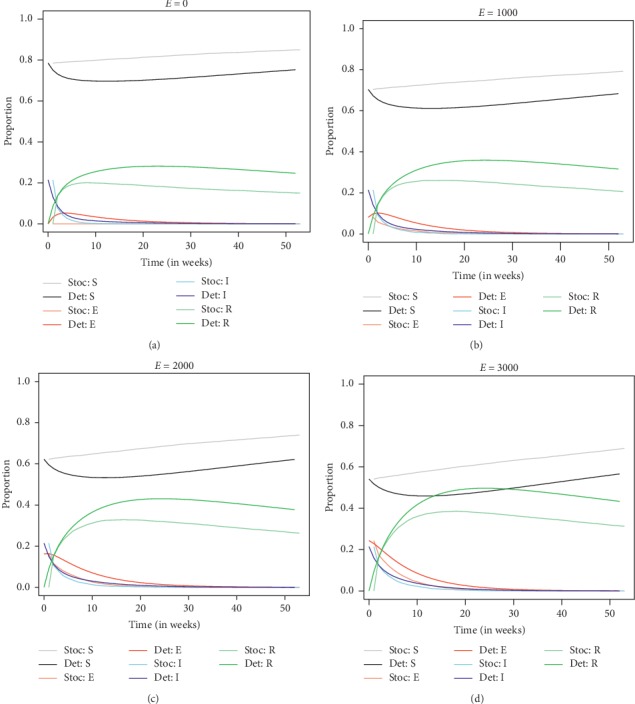
TB infection dynamics of deterministic and stochastic SEIR models with demography over time at varying initial exposed size (*λ*=*μ*=0.007;  *α*=0.2735;  *β*=0.5;  *ε*=0.1667). (a) Case 1: *S* = 9663, *E* = 0, *I* = 2643, *R* = 10. (b) Case 2: *S* = 8663, *E* = 1000, *I* = 2643, *R* = 10. (c) Case 3: *S* = 7663, *E* = 2000, *I* = 2643, *R* = 10 (d) Case 4: *S* = 6663, *E* = 3000, *I* = 2643, *R* = 10.

Both deterministic and stochastic SEIR models with demography were compared in terms of TB infection dynamics (Figure 3). One-way sensitivity analyses of the two SEIR models were conducted by varying the initial condition of the exposed compartment so as to ascertain its effect on the overall infection dynamics of TB in the Ashanti Region. Thus, only the initial condition of the exposed compartment was varied over time, while keeping all other initial conditions and parameters constant. The sensitivity analysis was performed using the initial conditions of the other compartments other than the exposed compartment of the entire region as a case study such that susceptibles = 9663, exposed = unknown, infectious = 2643, and recovery = 10. Therefore, parameter estimates were set as follows: birth and natural death rates: *λ*=*μ*=0.007, whereas exposed rate (*ε*)= 0.1667, recovery rate (*β*)  = 0.5, and infection rate (*α*)=0.2735.

It was revealed at the end of the infection period (52 weeks) that the stochastic model averagely overestimated the proportion of susceptible individuals relative to the deterministic SEIR model but underestimated the proportion of exposed, infectious, and recovered individuals at the varying initial size of the exposed compartment (Table 6). From the sensitivity analysis (summarized by Table 6 and Figure 3), it can be observed that increasing the size of the initial condition of the exposed compartment significantly decreases the proportion of susceptible individuals (with proportion of infected individuals relatively constant) but increases the proportion of recovered individuals at the end of the study period for both deterministic and stochastic SEIR models. The stochastic model generally overestimated the proportion of susceptible individuals over time as compared to the deterministic model but underestimated the proportion of individuals in the exposed, infectious, and recovered compartments.

### 3.5. Effect of the Model Parameters of SEIR on the Basic Reproductive Number

**Figure 4:**
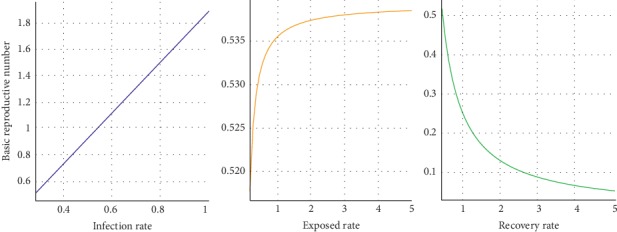
Effect of deterministic SEIR model parameters on the basic reproductive number.

The effect of the model parameters: infection rate (*α*), exposed rate (*ε*), and recovery rate (*β*) on the basic reproductive number (*R*_0_) were, respectively, investigated based on the deterministic SEIR model with demography (Figure 4). It was revealed that the SEIR model parameters have a varying effect on the basic reproductive number (*R*_0_). Increasing the exposed rate (*ε*) and the infection rate (*α*), respectively, increases *R*_0_ linearly and curvilinearly, which in turn increases the spread of TB. Nevertheless, the value of *R*_0_ decreases asymptotically towards zero (0) with an increase in the exposed rate.

### 3.6. Effect of Treatment Introduction at the Latent Period

**Figure 5:**
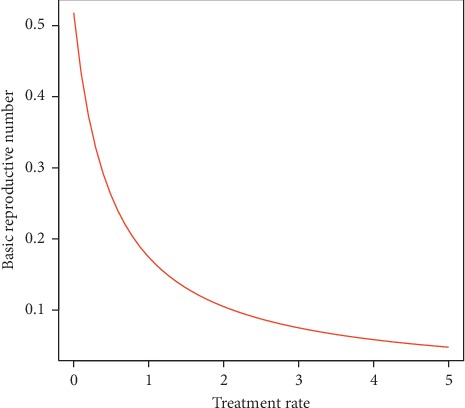
Effect of treatment introduced at the latent stage on the basic reproductive number.

The effect of initiating treatment at the exposed stage of tuberculosis infection on *R*_0_ was also explored (Figure 5). It was revealed that the initiation of treatment at the latent stage has great influence on the basic reproductive number. Increasing the rate of treatment at the exposed stage gradually decreased the reproduction number (*R*_0_), hence reducing the spread of TB by infected individuals as established by Theorem 1.

### 3.7. Numerical Simulation and Validation of Empirical Epidemic Thresholds

**Figure 6:**
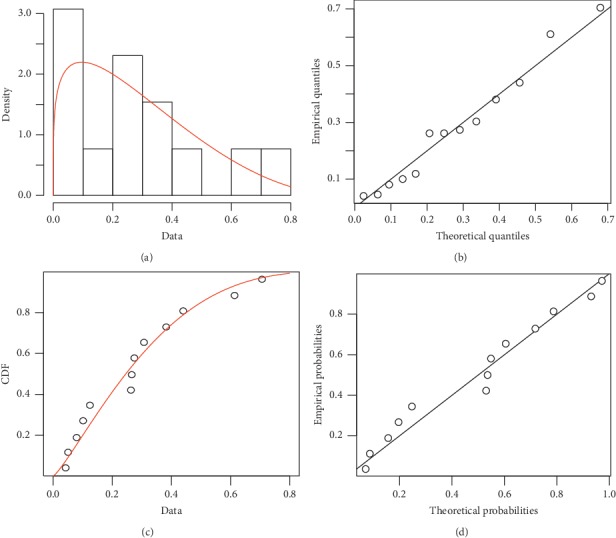
Distribution plots of fitted beta(1.223, 3.128) distribution to infection rates across the high-burden districts. (a) Empirical and theoretical densities. (b) *Q*-*Q* plot. (c) Empirical and theoretical CDFs. (d) *P-P* plot.

Numerical simulation was carried out to validate the empirical results of the various thresholds (basic reproductive number, herd immunity threshold, and Malthusian parameter) estimated in the study for the entire region. To improve on the various thresholds for the entire region, several probability distributions were fitted to the empirical infection rates across all districts (0.122, 0.441, 0.705, 0.048, 0.261, 0.306, 0.100, 0.081, 0.612, 0.381, 0.266, and 0.041) via maximum likelihood estimation. Beta-distribution best fitted the infection rates as compared to other continuous probability distributions based on their respective Akaike information criterion (AIC) values. The infection rates specifically followed beta(1.223, 3.128) probability distribution.  Figure 6 presents distribution plots of infection rates based on the fitted beta-distribution. It also suggests mathematically that the TB infection rate as a random variable appears to fit well with the beta-distribution for the sake of other inferential statistics such as Bayesian estimation.

To validate the empirical estimates of the overall infection rate and epidemic thresholds of the entire region, three different sampling schemes: bootstrap (random sampling with replacement), jackknife (leave-one-out sampling), and Latin Hypercube (stratified sampling scheme to improve on the coverage of the *k*-dimensional input space) were used to obtain 10000 simulated samples from beta(1.223, 3.128) distribution. Mean estimates of the overall infection rate, basic reproductive number, herd immunity threshold, and Malthusian parameter based on these sampling schemes were computed for comparison (Table 7). The bias of estimators based on the three sampling schemes was computed in order to determine the method that yielded better estimates of the true epidemic quantities for the entire region. It was revealed that the level of precision among the three sampling schemes was relatively the same, but the Latin Hypercube sampling scheme had a smaller bias in the estimates of the overall infection rate, basic reproductive number, herd immunity threshold, and Malthusian parameter of the entire region than estimates from bootstrap and jackknife sampling comparatively. These estimates of the epidemic thresholds (*R*_0_=0.533 < 1,  *ρ*=−0.073 < 0,  and *q*_*c*_=−0.877 < 0) suggest that the entire region is expected to have herd immunity against TB infection. Hence, tuberculosis infection cannot persist in the region in general, even though 2 of the districts are prone to endemic TB.

## 4. Discussion

The transmission of tuberculosis was previously investigated in only one high-burden district called Amansie West in the Ashanti Region of Ghana using a deterministic SEIR model [19]. However, the Ghana Health Service has categorized twelve districts as high-burden districts among a total of twenty-seven districts in the region, of which Amansie West is part. In this paper, we primarily expanded the previous work by Dontwi et al. [19] including their fitted epidemic model, by investigating TB infection among all the high burden districts in the region using the SEIR model with demography within deterministic and stochastic settings. The stochastic model overestimated the proportion of individuals that remained susceptible, exposed, and infectious and recovered from TB relative to its deterministic model over time. The disparity in the proportion at each compartment or disease stage may be attributable to either the large size of the infectious individuals or the effect of the other sources of variations such as demographic variability [9]. It was discovered from our models that 2 of the 12 districts had a greater chance of endemic TB based on empirical estimates of the basic reproductive number, herd immunity threshold, and intrinsic growth rate. The districts with endemic TB were Obuasi Municipal and Amansie West. This finding aligned with that of Dontwi et al., where they revealed a potential TB outbreak within the Amansie West district. Major reasons that increase the chance of TB disease within these two districts are mainly due to the general practices of the people through exploitation of resources of the surrounding lands such as farming as well as illegal mining and high congestion [19, 35, 36], resulting in lots of social contacts, immigration, and records of high number of new cases annually by TB programmes in these districts.

Nevertheless, estimates of the epidemic thresholds (*R*_0_=0.53 < 1,  *ρ*=−0.073 < 0,  and *q*_*c*_=−0.87 < 0) from bootstrap, jackknife, and Latin Hypercube sampling schemes for the entire region suggested that there may not be tuberculosis outbreak in the Ashanti Region since the probability of TB extinction was unity within the region as a whole; hence, the disease-free equilibrium point will be stable for the entire Ashanti Region. A similar result was found by Twumasi et al. where they discovered with a certain probability that TB-infected individuals can recover in the region via a discrete-time Markov model [37]. Nonetheless, approximately 12% and 14% of the study population in Obuasi Municipal and Amansie West district, respectively, need TB immunization to control the spread of the disease. Other studies also discovered that males in the region are more likely to contract TB due to social responsibilities of males, which require them to have more social contacts, thereby increasing the risk of TB exposure [38]. This implies that the current study can be extended to capture such demographic variations using at least multicohort epidemic models for different subgroups in the same population under study.

The sensitive analyses of both the deterministic and stochastic models were carried out to explore the effect of TB exposure on the overall infection dynamics. It was discovered from both models that increase in the TB exposure only decreased the proportion of susceptible individuals and increased the proportion of recovered individuals but had no significant effect on the proportion of infectious individuals over time, as also revealed from other studies [19]. Thus, not all exposed individuals will become infectious and even infectious people can recover from the disease with certainty at the acute stage. This also confirms that the number of exposed individuals can have a significant effect on the dynamics of tuberculosis in the Ashanti Region; hence, latent TB tests for exposure cannot be ignored as practically observed within most regions in the country due to under-reporting and underdiagnosis of TB cases in Ghana. Additionally, a marginal increase of the infection rate significantly caused a linear increase in the basic reproductive number (*R*_0_ > 1) in the region, while an increase in the exposed rate increased *R*_0_, but asymptotically below unity. However, an increase in the recovery rate effectively declined the basic reproductive number asymptotically towards zero. It was also revealed that the introduction of treatment at the latent stage of TB infection steeply decreased *R*_0_ towards 0. Chowell et al. and Mbogo et al. argued that increasing the infection rate implies increasing the number of infectious individuals, while increasing the exposed rate simply suggests decreasing the exposed or incubation period. Nonetheless, increasing the recovery rate means a decline in the infectious period [39, 40].

## 5. Conclusion and Recommendation

The SEIR model showed success in modelling infection dynamics of tuberculosis among high-burden districts in the Ashanti region of Ghana. Estimates of relevant epidemic thresholds (basic reproductive number, herd immunity threshold, and Malthusian parameter) and the probability of TB extinction within the entire region suggest that TB infection cannot be epidemic, and thus, it is certain to become extinct completely from the region. This implies further that with early diagnosis and treatment of TB, the prevalence of the disease can effectively be reduced over time within the region. However, approximately 12% and 14% of the study population in Obuasi Municipal and Amansie West districts, respectively, require TB immunization to control the spread of the disease since endemic TB is likely to occur in these two districts of twelve districts under study. Also, it was revealed that the number of exposed individuals, if left attended, can tremendously affect the number of completely susceptible individuals over time and the entire infection dynamics of TB. We recommend that the Ghana Health Service should find a good mechanism such as the Tuberculin Skin Test [41] to detect individuals who are exposed to TB at the early stages of infection due to under-reporting and underdiagnosis of TB cases in the region as well as its long-term effect when left undetected among exposed individuals. Finally, public health education and other symposiums must be organized on the prevalence of tuberculosis in the Ashanti Region, especially Amansie West district and Obuasi Municipal, as these two districts have a greater risk of encountering endemic TB. This will help to create a high level of awareness about the deadly nature of tuberculosis and the need to seek medical attention upon experiencing the signs and symptoms of the disease.

## Figures and Tables

**Algorithm 1:**
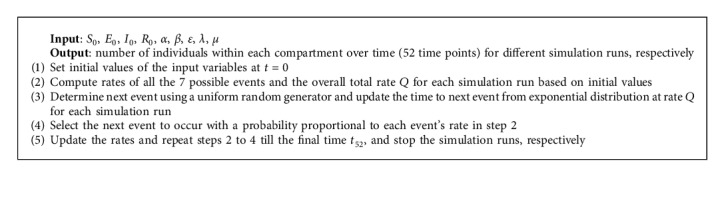
Pseudocode of Gillespie's SSA for the stochastic SEIR model with demography.

**Table 1:** The modelling scheme of the SEIR CTMC model with demography.

Event	Transition	Rate of occurrence within Δ*t*
Susceptible birth	*S*⟶*S*+1	*λN*
Susceptible death	*S*⟶*S* − 1	*μS*
Exposed	*S*⟶*S* − 1, *E*⟶*E*+1	*αS*(*I*/*N*)
Exposed death	*E*⟶*E* − 1	*μE*
Infection	*E*⟶*E* − 1, *I*⟶*I*+1	*εE*
Infectious death	*I*⟶*I* − 1	*μE*
Recovery	*I*⟶*I* − 1, *R*⟶*R*+1	*βI*
Recovered death	*R*⟶*R* − 1	*μR*

**Table 2:** Estimates of the infection rate among the high-burden districts.

Number	District	Total contact	Effective contact	Infection rate (*α*)
1	Adansi South	444	54	0.122
2	Asanti Akim North Municipal	2150	948	0.441
3	Amansie West	332	234	0.705
4	Mampong Municipal	790	38	0.048
5	Atwima Nwabiagye	440	115	0.261
6	Bekwai Municipal	455	139	0.306
7	Bosomtwe	570	57	0.100
8	Ejusu–Juaben Municipal	774	63	0.081
9	Obuasi Municipal	183	112	0.612
10	Offinso Municipal	21	8	0.381
11	Kumasi Metropolitan	3251	864	0.266
12	Sekyere South	243	10	0.041

**Table 3:** Estimates of the basic reproductive number, Malthusian parameter, and herd immunity threshold across districts.

Number	District	Basic reproductive number (*R*_0_)	Malthusian parameter (*ρ*)	Herd immunity (*q*_*c*_)
1	Adansi South	0.231	−0.123	−3.329
2	Asanti Akim North Municipal	0.835	−0.022	−0.198
3	Amansie West	1.158	0.041	0.136
4	Mampong Municipal	0.091	−0.15	−9.989
5	Atwima Nwabiagye	0.495	−0.073	−1.020
6	Bekwai Municipal	0.578	−0.060	−0.730
7	Bosomtwe	0.189	−0.130	−4.291
8	Ejusu-Juaben Municipal	0.154	−0.137	−5.494
9	Obuasi Municipal	1.334	0.020	0.118
10	Offinso Municipal	0.721	−0.038	−0.387
11	Kumasi Metropolitan	0.503	−0.072	−0.988
12	Sekyere South	0.078	−0.154	−11.825

**Table 4:** Estimates of the coefficient of the characteristics equation for disease-free districts.

District	*Y*	*Z*	*A*	*YZ* − *A*
Adansi South	0.6877	3.0725	0.00052	2.11291
Asanti Akim North Municipal	0.6877	3.0192	0.00015	2.07615
Mampong Municipal	0.6877	3.0847	0.00061	2.12074
Atwima Nwabiagye	0.6877	3.0492	0.00036	2.09657
Bekwai Municipal	0.6877	3.0418	0.00031	2.09154
Bosomtwe	0.6877	3.0761	0.00055	2.11489
Ejusu–Juaben Municipal	0.6877	3.0792	0.00057	2.11699
Offinso Municipal	0.6877	3.0292	0.00022	2.08295
Kumasi Metropolitan	0.6877	0.0484	0.00055	2.09580
Sekyere South	0.6877	3.0859	0.00062	2.12156

**Table 5:** Estimates of the coefficient of the characteristics equation for endemic districts.

District	*Y*	*Z*	*A*	*YZ* − *A*
Amansie West	0.6877	5.52 × 10^−3^	−0.07996	0.08375
Obuasi Municipal	0.6877	6.356 × 10^−3^	−0.07870	0.08310

**Table 6:** Population proportions of both deterministic and stochastic SEIR models at the end of the study period (52 weeks).

Type of model	Population proportions at the end of the study time				
	Exposed size	Susceptible (*S*)	Exposed (*E*)	Infectious (*I*)	Recovery (*R*)
Deterministic	0	0.751	7.12 × 10^−4^	2.86 × 10^−4^	0.248
	1000	0.681	8.28 × 10^−4^	3.38 × 10^−4^	0.318
	2000	0.619	8.23 × 10^−4^	3.41 × 10^−4^	0.380
	3000	0.563	7.63 × 10^−4^	3.2 × 10^−4^	0.436

Stochastic	0	0.850	0.000	0.000	0.150
	1000	0.794	9.71 × 10^−6^	4.55 × 10^−6^	0.206
	2000	0.737	1.92 × 10^−5^	9.52 × 10^−6^	0.263
	3000	0.681	2.96 × 10^−5^	1.44 × 10^−5^	0.319

**Table 7:** Estimation of overall infection rate and other epidemic thresholds of the entire region using different sampling sampling schemes.

Sampling scheme	Parameters	Estimates	Bias
Bootstrap	*α*	0.28278	0.00928
	*R*_0_	0.53529	0.01729
	*ρ*	−0.07298	0.00102
	*q*_*c*_	−0.86825	0.06225

Jackknife	*α*	0.28279	0.00929
	*R*_0_	0.53531	0.01731
	*ρ*	−0.07297	0.00103
	*q*_*c*_	−0.86807	0.06243

Latin hypercube	*α*	0.28148	0.00798
	*R*_0_	0.53282	0.01482
	*ρ*	−0.07331	0.00069
	*q*_*c*_	−0.87682	0.05368

^*∗*^Empirical estimates: *α*= 0.2735, *R*_0_= 0.518, *ρ*=−0.074, and *q*_*c*_=−0.9305.

## Data Availability

The secondary data (in Microsoft Excel Worksheet) used for the study will be made available upon request from the corresponding author.
